# Protein Source and Muscle Health in Older Adults: A Literature Review

**DOI:** 10.3390/nu13030743

**Published:** 2021-02-26

**Authors:** Christianto Putra, Nicolai Konow, Matthew Gage, Catherine G. York, Kelsey M. Mangano

**Affiliations:** 1 Center for Population Health, Department of Biomedical and Nutritional Sciences, University of Massachusetts Lowell, Lowell, MA 01854, USA; christianto_putra@student.uml.edu (C.P.); Catherine_York@student.uml.edu (C.G.Y.); 2 UMass Movement Center, University of Massachusetts Lowell, Lowell, MA 01854, USA; Nicolai_Konow@uml.edu (N.K.); Matthew_Gage@uml.edu (M.G.); 3 Department of Biological Sciences, University of Massachusetts Lowell, Lowell, MA 01854, USA; 4 Department of Chemistry, University of Massachusetts Lowell, Lowell, MA 01854, USA

**Keywords:** dietary protein, pea, older adult population, muscle protein synthesis, sarcopenia

## Abstract

Research shows that higher dietary protein of up to 1.2 g/kg_bodyweight_/day may help prevent sarcopenia and maintain musculoskeletal health in older individuals. Achieving higher daily dietary protein levels is challenging, particularly for older adults with declining appetites and underlying health conditions. The negative impact of these limitations on aging muscle may be circumvented through the consumption of high-quality sources of protein and/or supplementation. Currently, there is a debate regarding whether source of protein differentially affects musculoskeletal health in older adults. Whey and soy protein have been used as the most common high-quality proteins in recent literature. However, there is growing consumer demand for additional plant-sourced dietary protein options. For example, pea protein is rapidly gaining popularity among consumers, despite little to no research regarding its long-term impact on muscle health. Therefore, the objectives of this review are to: (1) review current literature from the past decade evaluating whether specific source(s) of dietary protein provide maximum benefit to muscle health in older adults; and (2) highlight the need for future research specific to underrepresented plant protein sources, such as pea protein, to then provide clearer messaging surrounding plant-sourced versus animal-sourced protein and their effects on the aging musculoskeletal system.

## 1. Introduction

Protein is a critical macronutrient for maintenance of normal bodily functions. Required daily protein intake varies by age, sex, and degree of daily activity, but is critical to maintain muscle mass and strength throughout an individual’s lifespan [1]. Muscle mass and muscle strength are positively correlated, independent of age and gender [2]. Generally, greater lean mass is associated with better overall health [3] and prevention of functional declines associated with aging [4]. However, after the age of 30 year, muscle mass declines at a rate of 0.3 to 0.8% per year [5]. The reduction in lean mass is attributed to the reduction in number, and to some extent area size, of myofibers [6]. Factors affecting muscle mass include age-related muscle atrophy, decreased mitochondrial function, increased oxidative stress, impaired satellite cell function, and inflammation [7]. In addition, muscle strength is a major predictor of disability and all-cause mortality in older adults [8,9] with some research suggests muscle strength is a more meaningful predictor of health outcomes compared to muscle mass [9]. Concomitant with muscle atrophy, muscle strength also declines with age, especially after 50–60 year [10,11] at a rate of 2 to 4% [12] or greater in the lower limbs every year [13]. Therefore, it is imperative to prevent age-associated losses in muscle mass and strength to enhance overall performance throughout the lifespan.

Sarcopenia, or age-related musculoskeletal decline, is characterized by: (1) low muscle strength (tested via grip strength and chair stand test); (2) low muscle quantity or quality (confirmed via dual-energy x-ray absorptiometry (DXA), computed tomography (CT), or magnetic resonance imaging (MRI), etc.); and (3) low physical performance (assessed via gait speed, 400 m walk test, etc.) [14]. The potential etiology of sarcopenia is a combination of declines in physical function, loss of muscle mass, increased inflammation, altered hormone balance, inadequate nutrition intake, and anabolic resistance [15]. Sarcopenia currently affects 6–19% of individuals worldwide over the age of 60 year [16]. Individuals with sarcopenia are at an increased risk of falls, fractures, decreased quality of life, cardiovascular disease, respiratory disease and cognitive impairment [14]. Risk of sarcopenia is dependent upon age, ethnicity, living conditions, and pre-existing medical conditions [14]. The condition was estimated to cost $18.5 billion of direct health care costs in the year 2000 [17] and individuals with sarcopenia will endure double the cost of hospitalization compared to those without sarcopenia [18]. Due to the increasing number of older adults living longer it is imperative to identify solutions to prevent and treat this debilitating disease.

Currently, there are no approved medications for the treatment of sarcopenia. The standard of care for hospitalized or institutionalized older adults with frailty or sarcopenia includes participation in resistance-training and weight-bearing exercises [19,20,21]. However, the transition from a hospital bed to rehabilitation is challenging, as many patients are unable to engage in physical activity due to lack of exercise equipment, social support, and self-motivation to exercise [22]. Diet is a modifiable lifestyle factor that may serve a strong role in preventing and treating sarcopenia [21]. For example, greater protein intake is linked to improvements in muscle mass and strength [23,24]. However, the current recommended daily allowance (RDA) from the World Health Organization (WHO) [1] and National Academies of Sciences [25] of 0.8 g/kgbodyweight/day for adults may not be sufficient to combat sarcopenia in older adults. Recent evidence suggests that protein intake closer to 1.2–1.5 g/kgbodyweight/day, in combination with adequate exercise, is more beneficial in preventing age-related declines in muscle mass and strength, improving health status, and reducing the risk of early mortality [3,26,27,28]. Despite growing evidence that a higher RDA for dietary protein is beneficial to older adults, there are numerous barriers to reaching this dietary goal. Older adults face an increased risk of protein-energy malnutrition due to social isolation [29], lack of appetite, and potential issues with mastication [30]. In addition, protein increases satiety, and therefore, older adults are less likely to meet their protein requirements due to feelings of fullness and meal skipping. Therefore, it is critical to provide sustainable, nutrient-dense protein sources that will maximally enhance muscle mass and strength to reduce the risk of developing sarcopenia in this at-risk group.

A consensus on whether protein sources differentially affect the progression of sarcopenia has yet to be determined. Animal and plant protein-containing foods differ in their amino acid content [31], absorption kinetics, and nutrient to food matrix interaction [32]. Protein quality and digestibility are distinguishing features between animal and plant proteins. Traditionally, whey protein, has been commonly used for dietary protein supplementation because of its high digestibility and complete amino acid profile [33]. Meanwhile, there is growing clinical and consumer market interest in vegetarian and vegan diets due to their potential health benefits, environmental sustainability, and ethical issues surrounding raising animals for the sole purpose of consumption [34]. Historically, soy protein has been the go-to plant-based protein due to its near complete essential amino acid (albeit low methionine) profile; however, pea protein is gaining popularity among consumers due to recent advances in industry produced meat alternative products. Despite their growing consumer popularity, there is little scientific research on whether plant protein alternatives are effective in preventing age-associated muscle losses compared to animal protein counterparts. Therefore, the primary objectives of this review are to:

(1) Review current literature from the past 10 years evaluating whether specific source(s) of dietary protein provide maximum benefit to muscle health in older adults; and
(2) to provide messaging surrounding plant-sourced versus animal-sourced protein and their effects on musculoskeletal aging, while highlighting the need for future research specific to underrepresented plant protein sources, such as pea protein.

## 2. Discussion of the Current Status of Knowledge

### 2.1. Protein Quality and Digestibility Differs by Protein Source

Skeletal muscle mass is regulated by the balance between the rate of muscle protein synthesis (MPS) and the rate of muscle protein breakdown (MPB), which are both regulated by a variety of factors. During muscle accrual, the rate of MPS is greater than MPB. It is generally accepted that dietary protein and physical activity stimulate MPS either independently [35,36,37,38] or interdependently [39,40]. Muscle protein synthesis is a process dependent on postprandial availability of essential amino acids (EAAs) [31]. The amount and diversity of EAAs to meet human nutritional needs is defined as protein quality [41]. The most widely adopted indexes of protein quality are the Protein Digestibility Corrected Amino Acid Score (PDCAAS) and the Digestible Indispensable Amino Acid Score (DIAAS). Protein food sources vary widely in their PDCAAS and DIAAS scores. For example, milk, whey, egg, casein, and soy protein isolate scored 1.00 (highest possible score) on the PDCAAS. In comparison, cooked yellow pea or *Pisum sativum*, pea protein concentrate (NUTRALYS^®^), and pea protein isolate (NUTRALYS^®^, manufactured by Roquette, Lesterm, France ) have a PDCAAS of 0.67 [42,43], 0.893 [44] and 0.93 [45], respectively. When compared using DIAAS, whey protein isolate, whey protein concentrate, soy protein isolate, and pea protein concentrate have a DIAAS of 100, 107, 84, and 62, respectively [46]. Therefore, animal and soy-based protein foods, generally have a higher protein digestibility score compared to pea-sourced protein, regardless of the scoring method used.

In addition, protein digestibility and absorption kinetics will dictate the amount of immediate EAA available to stimulate MPS response [47]. Faster protein digestion and absorption typically lead to a more acute MPS response and a higher peak compared to slower absorbing proteins [48]. Depending on the processing method and the presence of “antinutritional factors”, it is generally shown that plant-based proteins have lower digestibility compared to animal-based proteins [43]. Antinutritional factors are food components or compounds such as tannins, phytate, oxalate, saponins, lectins, alkaloids, protease inhibitors, cyanogenic glycosides [49] inside the food matrix that interfere with the absorption of certain nutrients. An example of an antinutritional factor that interferes with protein absorption is Bowman-Birk trypsin inhibitor, which can be found in foods such as soybean grits, soymilk, soy isolate, and soy protein concentrate [50], which inhibits the enzymatic action of pepsin and trypsin in the gut [51]. Certain antinutritional factors can be inactivated through cooking to improve protein digestibility [52]. Also, it is important to note that ingested plant protein (e.g., soy protein) increases protein oxidation [53], which suggests amino acids from plant protein is used for the production of urea and therefore, less amino acids are available to stimulate MPS [43,54,55]. Due to the differences in digestibility and quality among protein sources, it is important to take these factors into consideration when developing nutrition interventions to prevent age-associated muscle losses.

### 2.2. Muscle Response to Animal Versus Plant Protein Sources May Not Differ at Higher Protein Intakes

Research comparing the anabolic properties of the various plant- and animal-based protein sources is important to determine whether specific nutrition regimens can be formulated to maximize the muscle health of older adults. Differences between milk protein, particularly whey, versus soy protein have been well studied in adults and young adults. Table 1 illustrates selected clinical trials from the past 10 years and some notable older studies that examined the effect of differing sources of dietary protein on muscle outcomes. It has been shown that soy protein ingestion results in a lower MPS compared to whey [53,56] during both rested and post-exercise conditions. Volek, et al. [57] conducted an experiment on 63 randomized healthy adults that performed whole body resistance training program and consume isocaloric supplements containing carbohydrate, whey (24 g), or soy protein (24 g) for 9 months. The authors show that whey supplementation significantly increased fasting plasma leucine concentration and lean body mass gains compared to carbohydrate and soy protein supplementation regimens. However, other studies suggest when higher protein supplementation doses are consumed (>30 g) in combination with an exercise regimen, muscle outcomes are similar across protein subtypes. For example, a study where omnivorous participants followed an 8-week progressive, non-linear resistance training protocol in addition to supplementation with 48 g of either rice or whey protein isolate showed that there was no difference in body composition and exercise performance [58]. Similarly, other studies with isonitrogenous supplementation of 33 g soy or whey protein resulted in similar increases in muscle mass after exercise training in omnivorous participants [59,60]. A meta-analysis conducted by Messina, et al. [61] concluded that soy protein supplementation did not yield any differences in lean body mass and strength in response to resistance exercise training in healthy omnivorous adults between the age of 18 to 70 years compared to whey protein. However, the authors noted that the independent influence of age or sex could not be identified, and, thus, they recommend more research, specifically among older individuals. In a diet study of whole foods without an exercise intervention, Campbell, et al. [62] show that older women who consumed an omnivorous diet (1.0 ± 0.08 g/kgbodyweight/day of protein) gained more lean body mass over 12-weeks compared to those consuming a lacto-ovo-vegetarian diet (0.78 ± 0.1 g/kgbodyweight/day of protein). In subsequent research [63], they found that the observed difference was attenuated by increasing the amount of protein consumed by the lacto-ovo-vegetarian diet to 1.15 g/kgbodyweight/day. Although these findings do not directly compare animal versus strictly plant protein diets, the results suggest that ingestion of higher amounts of total dietary protein may overcome the different properties of animal versus plant proteins and their influence on muscle outcomes.

It is important to note that high protein intake in older populations may not be suitable for individuals with reduced renal function due to increased filtration burden from greater protein intake. Higher protein consumption has been shown to exacerbate a declining renal function under conditions of modestly impaired renal function [73]. Older adults with severe kidney disease (estimated Glomerular Filtration Rate < 30 mL/min) and who are not on dialysis, are recommended to limit their protein intake until they receive dialysis [27]. More importantly, initial onset of chronic kidney disease is often asymptomatic. Precautionary measures (e.g., routine serum creatinine or blood urea nitrogen test) may be needed in individuals with comorbidities before engaging in a high protein diet regimen [74].

Although literature suggests that in older adults, consuming adequate protein may be the strongest determinant of healthy muscle outcomes [75,76,77], and type of protein may be less important, meeting higher dietary protein requirements can be challenging. Older adults have been shown to eat less protein due to social isolation [29], lack of appetite, and problems with dentition [30]. In addition, protein increases satiety [78] and may reduce overall energy intake among older adults. Therefore, supplementation with high-quality protein may help to circumvent these issues. However, the logical question remains, is there an optimal source of supplemental protein for older adults? A study by Gorissen, et al. [79] demonstrates the stark differences between types of supplemental proteins, where whey, casein, soy, and pea protein isolates have 43%, 34%, 27%, and 30% of varying EAAs of the total protein, respectively. The authors conclude that for an adult to consume 2.7 g of leucine, a strong determinant of MPS, or 10.9 g essential amino acids, they would have to ingest ~32 g of whey protein, ~47 g of casein protein, ~55 g of soy protein, and ~48 g of pea protein. Due to the differences in EAA profile among protein subtypes, testing muscle response to these supplemental proteins is imperative among older adults to determine if an optimal source exists and can overcome barriers to meeting recommended total protein intakes.

### 2.3. Supplementation with Leucine and Essential Amino Acids May Benefit Older Adults with Low Protein Intake

Various amino acids, such as β-alanine, l-glutamine, l-arginine, l-leucine and its bioactive form (hydroxymethyl butyrate) have been studied for nutritional management of sarcopenia because they are anti-catabolic and anabolic in nature [80]. For example, studies have shown that supplementation of β-alanine [81,82,83,84] may prevent and delay the progression of sarcopenia by addressing the systemic depletion of carnosine (β-alanyl-l-histidine), a dipeptide that acts as a pH buffer and is predominantly found in skeletal muscle. However, of these substrates, leucine is the most well studied, and requires greater understanding regarding adequate dosing and short-term versus long-term effects on muscle health [80]. Therefore, this review will discuss research surrounding the effects of leucine supplementation in older adults with low protein intake.

Certain EAAs elicit more MPS, notably leucine, compared to others by activating the mammalian target of rapamycin (mTOR) signaling pathway [85]. It is important to note that mTOR activation is the key regulator of human MPS in response to increased EAA availability [86]. Leucine not only activates MPS through mTOR complex 1 (mTORC1) [85], but also activates MPS through a mTORC1-independent processes [87,88]. But, it is important to note that leucine alone does not stimulate MPS; and actually require other amino acid to sustain anabolism [89]. For comparison, whey, casein, egg, soy, and pea protein isolate have 8.6 g, 5.8 g, 3.6 g, 5.0 g, 5.7 g of leucine per 100 g of food, respectively [79]. Research has shown that MPS response to suboptimal doses of protein (6.25 g of whey protein), but with supplementation of leucine (to reach a total of 3.0 g of leucine in dose) is similar to ingestion of 25 g of whey protein in healthy young men [64]. Thus, food or supplements that are highest in leucine, like whey protein, could be used to maximize the potential MPS response. It may also be possible to spike plant protein supplementation with adequate leucine content to match anabolic responses seen with whey protein alone. For example, supplementation of wheat protein with leucine to match the amount of leucine content in whey protein resulted in an equalized rate of MPS in adult rats [90]. A study in older adults consuming lower protein meals (0.81 ± 0.04 g protein/kg/day) supplemented with 4 g of leucine per meal 3 times a day for 2 weeks demonstrated improved muscle protein synthesis, acutely [65]. Similarly, Engelen et al. [66] demonstrated that consumption of branched chain amino acid (BCAA) spiked soy protein significantly increased whole-body protein synthesis in patients suffering from a chronic wasting disease (e.g., chronic obstructive pulmonary disease (COPD)) when compared with soy protein alone. Moreover, a recent clinical trial reported that leucine requirements that was determined by indicator amino acid oxidation method (IAAO) with L-1[1-13C] phenylalanine as the indicator in healthy older adult males and females >60 y, reported that their requirement was 77.8 mg/kgbodyweight/day for male and 78.2 mg/kgbodyweight/day for female which is double that of the current international RDA for leucine (39 mg/kgbodyweight/day) [91]. Overall, as older adults typically consume below the optimal EAA dosage at each meal (due to overall low protein consumption and/or lack of high-quality protein intakes [21,92]), supplementation with a protein high in leucine may be needed to assist in forestalling age-related muscle loss.

Although supplemental leucine doses of 3.0 g have been shown to increase MPS, there are observed limits to how efficacious leucine can be towards MPS. Moore et al. [93] tested the leucine dose-response relationship and threshold theory through a dose-response study using 0 g, 5 g, 10 g, 20 g, and 40 g of albumin in exercised young men on MPS outcomes where the blood amino acid level after exercise peaked around 1-h post-ingestion. The authors showed that MPS increased in a dose-dependent manner in response to dietary protein ingestion and reached the maximum MPS response at 20 g of albumin (which contains approximately 1.7 g of leucine, 0.25 g protein/kgbodyweight); and these results are supported by two other studies [94,95]. Further, Moore et al. [93] observed no significant additional increase in MPS at higher doses (40 g of albumin, 3.4 g of leucine). They noted that leucine oxidation significantly increased above the level of maximum MPS, which suggests that the additional leucine was unable to be utilized [93,95]. Overall, the maximal MPS response in older adults was observed at ~35–40 g of protein post-exercise [96] and 20 g at rest [93]. At the same time, the benefit of increased leucine content diminishes as the amount of total protein increases [97,98] and supplying excess leucine, or any essential amino acids for that matter, will result in plateauing of the MPS response [93,95,99,100]. In addition, the long-term effect of leucine supplementation on muscle health remains unclear. Supplementation of leucine in exercised older adults [101] could increase MPS response up to 24-h after supplementation; however, the acute increase in MPS was not associated with increases in lean body mass [102]. A 3-month study in healthy older men (71 ± 4 year) [67] and a 6-month study in diabetic older men (71 ± 1 year) [68] showed supplementation with 7.5 g leucine per day did not increase skeletal muscle mass or strength. Similarly, in a separate study of 21 adults ≥ 65 year, supplementation with 15 g of EAAs twice a day during bed rest resulted in improvements in MPS to a similar level observed pre-bedrest; however, the augmentation of MPS did not prevent muscle loss as measured by DXA [69]. Another exercise and leucine-rich-EAA supplementation (3 g twice a day) study among 155 sarcopenic older women (≥75 year) showed significantly improved walking speed after a comprehensive training program twice a week for 3 months, but neither lean mass nor strength improvements were observed [70]. Therefore, the long-term benefits of leucine supplementation on muscle health and functionality remains to be determined. Even though higher total protein intakes may compensate for varying leucine intake in adults meeting the dietary guidelines for protein, older adults with chronically low protein intakes may be a subgroup that would additionally benefit from leucine supplementation.

### 2.4. Associations between Whole Food Sources of Protein and Muscle Outcomes in Humans

With studies demonstrating short-term MPS response differs by protein quality, digestibility, and leucine content, there is a need to examine the relation of usual protein intake with muscle health among older adults [43]. In addition, it is important to examine the effects of plant protein sources on muscle within the context of usual dietary patterns as there is increasing interest in plant-based diets due to their known healthful effects on metabolic health [103]. Consuming greater amounts of protein, regardless of source, could compensate for the lower EAA content of plant-sourced proteins. In addition, incomplete proteins can be combined in the diet to form complete EAA profiles. For example, grain proteins are typically lower in lysine and higher in methionine. In contrast, beans are typically lower in methionine, but higher in lysine. When these plant-protein foods are combined together, grain and bean proteins form an EAA profile that resembles animal protein [43]. However, postprandial blood EAA concentration following ingestion of high quality plant protein blends was not the same as whey protein when matched to its leucine content [104]. Therefore, protein quality and digestibility indexes such as PDCAAS and DIAAS do not provide information regarding the true anabolic effect of specific plant protein sources on body tissues compared to animal source counterparts. A systematic literature review by van Vliet, Burd and van Loon [43] highlighted that animal protein is generally, at an isonitrogenous amount, more superior in promoting muscle protein synthesis and leads to greater muscle mass compared to plant-sourced protein (e.g., soy and wheat). The authors show that plant-based protein offers equal benefits compared to animal-sourced protein at greater amounts of ingestion. However, this might not be the case in certain populations. In the past, studies showed that vegetarians tend to consume less daily dietary protein compared to their omnivorous counterparts and result in lower muscle mass [62,105,106]. However, it is difficult to determine whether the differences in muscle mass were due to protein-specific dietary pattern or difference in overall protein intake.

Epidemiological studies on the associations of dietary source of protein with measures of muscle from the past 10 years are shown in Table 2. Data from the Framingham Offspring Cohort demonstrates that total protein intake and animal protein intake, but not plant protein, are associated with muscle mass as measured by DXA in older adult men and women [107]. Specifically, higher leg lean mass was observed among participants in the highest quartile of animal protein intake compared to the lowest quartile. In the same cohort, higher total protein and animal protein intake, but not plant protein, were suggested to be protective against loss of grip strength over 6 years among adults over the age of 60; interestingly, this prospective association was not observed in adults <60 year [108]. When using dietary pattern methodology specific to protein intake, considering all other nutrients and foods consumed in combination with protein intake, no associations between source of protein intake, appendicular lean mass and quadriceps strength were observed, when total protein intake was above the RDA [109]. Chan, et al. [110] showed that quartiles of total protein intake (Q1: 69.2 ± 28.6; Q4 83.3 ± 37.0 total protein g/day) and quartiles of animal protein intake (Q1: 37.1 ± 21.1; Q4: 46.8 ± 29.1 animal protein g/day) were not associated with changes in physical performance nor lean mass over 4 years. In contrast, the authors noted that a diet low in plant-based protein (Q1: 32.1 ± 13.9; Q4: 36.5 ± 19.7 plant protein g/day) was associated with higher muscle loss compared with those in the highest quartile of plant-protein intakes. The different outcomes observed between this study of Chinese adults and studies in cohorts with Western dietary patterns, may be due to differences in type and amount of plant protein consumed, and other intakes of nutrients that may influence muscle such as vitamin D, folic acid, and antioxidants. In addition, there is a need to standardize the protein dosage measurement (e.g., comparing g/kgbodyweight/day of each protein subtype) to further understand the differential impact between animal versus plant-sourced protein on muscle health parameters. Overall, these studies suggest that source of dietary protein may not matter as long as older adults are meeting the RDA for protein, habitually.

### 2.5. Increased Market Demand for Plant-Based Protein Supplements and Foods Demands Further Research in Humans

The U.S. protein supplement industry has a market size of $14 billion with a 67.9% share of the overall revenue in 2018 from animal-based protein [33]. The plant-based segment is expected to grow the fastest with projected compound annual growth rates (CAGR) of 8.6% from 2020 to 2027 with an estimated market share of $11.05 billion in 2027; and soy protein remains the leader in this segment. For millions of health-conscious, vegetarian and vegan adults, soy products such as tofu and soy milk are an important source of dietary protein [111,112,113]. The popularity of soy protein is partially due to its higher protein content and quality compared to other legumes, and its relatively similar digestibility and EAA profile to animal protein [44]. Due to soy protein’s popularity, and EAA content, its impact on health is often compared to that of animal protein. Despite being the leader in the segment of plant protein, market demand for soy-based protein is beginning to show a decline in popularity due to perceived consumer concerns surrounding allergens, phytoestrogens, and genetically modified organisms (GMOs) [33]. To-date, a search engine/Google search with the key phrase “plant protein” shows that the first 6 out of 8 protein powder supplements listed for sale contain pea protein isolates as their primary ingredient. Unlike soy protein, pea protein is: (1) less of an allergen risk, (2) does not contain phytoestrogens, and (3) not genetically modified [114].

In terms of EAAs profile, leucine content, its limiting amino acid, pea protein is relatively similar of that soy protein. The essential amino acid content of pea and soy protein are 37% and 38% of the total protein content, respectively [43]. The leucine content of pea and soy protein contain 7.8% and 8% of leucine of the total protein respectively [43]. While methionine is the first limiting amino acid in soy protein, the first limiting amino acid in pea protein is methionine and cystine [45].

The booming industry of meat product-alternatives is predicted to reach $140 billion over the next decade [115] with pea protein as the fastest-growing source of protein in that segment. The soaring popularity of Beyond Meat^®^ (Beyond Meat, El Segundo, CA, USA), an alternative meat product made from pea protein isolate, canola oil, various seasonings and additives, had a market value of $13.4 billion in 2019 [116]. With increasing demand from health-conscious consumers for pea protein-sourced products and increasing popularity of veganism/vegetarianism, pea protein could quickly become a growing staple in the typical American diet. However, as described above, scientific evidence evaluating muscle tissue response to varying protein sources is inconclusive, and studies examining plant protein sources other than soy, such as corn protein isolate [117] and potato protein isolate [118], especially in older populations, are limited. Therefore, research testing the differential association and/or effects of milk protein, whey and casein, soy, and pea protein on muscle outcomes in older adults is needed to support or dissuade use of these products for musculoskeletal gains.

### 2.6. Limited Research in Humans Assessing the Role of Pea Protein in Muscle Health

Despite the increasing market demand for pea protein, there are limited studies examining their effect on muscle, and to our knowledge, there are no studies in the older adult population. A study in 1970 by Bell and Youngs [119] studied the growth of weaning male mice fed with either pea protein concentrate, defatted whole egg, fish protein concentrates, or casein. In addition, they performed nutrient content analysis and found that the amino acid percentages between pea protein concentrate and fish protein concentrate are remarkably similar except that pea protein concentrate is much lower in methionine and higher in cystine. Following the 14 days of feeding, the authors found that at 10% protein of the macronutrient (other macronutrient composition was not disclosed), the pea protein concentrate protein-sourced fed mice grew sub-optimally (measured by bodyweight) compared to the other groups (casein, defatted whole egg, and fish protein concentrate). The pea protein group weighed on average ~6.5 g while other groups including the pea protein group supplemented with methionine, weighed ~10 g. In addition, they calculated the feed/weight gain ratio where the pea protein group was 7.0 while others ~5.0. Only after supplementing the 10% protein diet with methionine or adding feed containing 15% of pea protein concentrate in their diet, the growth of the mice was similar to mice in other protein groups. The finding by Bell and Youngs [119] was further supported by Martínez, et al. [120] where male Swiss albino mice fed with pea sourced protein (raw pea) showed stunted growth and low utilization of different nutrients compared to casein fed group. The observed poor outcomes in the pea protein fed mice were likely due to the presence of antinutritional factors (e.g., lectins) in the feed that inhibited nutrient absorption, and related to the poor content of *S*-amino acids in pea protein. In a study of untrained omnivorous human males between the ages of 18 to 35 years, participants were fed 25 g of whey (*n* = 54) protein twice a day or 25 g of pea protein isolate (NUTRALYS^®^) (*n* = 53) twice a day or a maltodextrin placebo (*n* = 54) for 12-weeks in combination with a resistance training program. At the end of the intervention, the authors found that biceps brachii muscle thickness increased by 20.2%, 15.6%, and 8.6% for pea, whey, and placebo, respectively [71]. In another smaller study, omnivorous men (*n* = 8) and women (*n* = 7) were randomized to whey (24 g) or pea (24 g) (True Nutrition, Vista, CA, USA) protein supplementation each before and after high-intensity functional training and between meals once a day on non-training days [72]. Overall, with 8-weeks of high-intensity functional training, the participants gained significant improvement in muscular strength in both groups. There were no differences in strength, body composition (bioelectrical impedance analysis), nor muscle thickness (at the midsection of rectus femoris measured using ultrasound imaging) between whey and pea protein supplemented groups. The lack of difference in muscle strength and body composition may be due to the relatively short 8-week training duration on already trained participants, again suggesting protein supplementation may only be beneficial to untrained, and/or protein deplete adults. It is worth noting that the whey protein and pea protein groups consumed relatively high protein consumption at 150.7 g/d and 129.9 g/day of protein, respectively which potentially masked the differential effect of between the two type of protein. These studies also provide little understanding on the effect of pea protein on older adults as they were conducted in younger groups (aged 13–35). In addition, further limitation to interpretation of these studies is due to lack of data on participants’ total dietary protein intake at baseline. Therefore, it is possible that lack of information on baseline protein intake may confound the presented results and future, well-designed studies are needed in this area.

## 3. Future Directions and Conclusions

Older adults and clinically compromised individuals are at risk for sarcopenia and protein malnutrition. A tailored nutrition approach to provide adequate protein (1.2 g/kgbodyweight/day), from a mix of sources, and supplementing sufficient leucine may offset these risks. Among older adults with chronic low protein intakes, a protein or leucine supplementation may be warranted to augment inadequate daily protein intake. There are a wide variety of protein beverages available in the market with varying sourcing of protein, such as whey, soy, and pea-based protein. Unlike pea protein, whey and soy-based proteins have been studied extensively for the past decade and have shown no differences in LBM and the strength in response to resistance exercise training in adults. This review highlights the limited number of studies conducted evaluating the effect of pea protein on muscle mass, strength, and function, despite its increasing popularity in the market. Essential amino acids such as leucine are critical in improving muscle protein anabolism, muscle size, and function, which is necessary in older populations. Therefore, future research is needed to evaluate the effect of pea protein with and without additional leucine, or its limiting amino acid, supplementation on muscle physiology and performance, primarily in the older adult population. In addition, a long-term trial with a practical approach to providing a high protein diet with defined health outcomes in various population groups is needed to establish which dietary protein source would be more beneficial; especially in the context of barriers older adult populations commonly face in meeting their protein needs.

It is important to review total protein intakes and protein sources within the context of the whole diet. The effect of dietary protein on muscle health in the context of other dietary factors should be examined as other nutrients have been shown to influence muscle, such as vitamin D, omega-3 fatty acids, antioxidants, acid-base diets, magnesium, and probiotics [21]. For example, studies have identified the pathway in which vitamin D supports skeletal muscle health [121]. Yet supplementation with vitamin D in states of deficiency yield conflicting results related to prevention of sarcopenia [121]. However, these nutrients are outside the scope of the current review paper. Therefore, we recommend a multi-arm randomized controlled clinical trial of protein supplementation in omnivores, vegetarians, and vegans while controlling for amount of baseline total protein intake could better explain the muscle effect of protein supplementation on each dietary pattern. Further, a large epidemiological study is needed to examine the association between animal versus plant protein on muscle outcomes among protein insufficient consumers to assess if the importance of protein source is magnified at lower total dietary protein compared to populations with higher total dietary protein. In addition, research that examines the influence of different protein sources on the development of sarcopenia across the lifecycle would contribute greatly to the advancement in prevention and treatment of sarcopenia and musculoskeletal aging. Lastly, future research is needed to better understand the role protein sources on the intersection of muscle and metabolic health.

In conclusion, providing tailored protein recommendations to older adults at nutritional risk may help slow the development of sarcopenia and, subsequently, provide a better quality of life. Current literature suggests recommendations should be targeted towards consuming enough total protein daily, especially in populations prone to malnutrition, through dietary protein or leucine supplementation. Further research evaluating the impact of various protein sources on muscle health is needed, with particular emphasis on lesser-studied plant proteins, such as pea, corn, potato, hemp or rice protein.

## Figures and Tables

**Table 1:** The effect of dietary protein supplementation by source and essential amino acid content on muscle outcomes in clinical trials.

Duration	Population, *n*	Exposure or Intervention	Amount of Protein	Outcome Measure	Results	References
3 h	Men Mean age 23 *n* = 18	Unilateral resistance training Whey vs. casein vs. soy	21.4 g, 21.9 g, or 22.2 g of protein, respectively	MPS	Relationship between protein intake and MPS are dose and protein source-dependent under rested and post-exercise conditions	[56]
4 h	Men Mean age 71 years *n* = 30	Unilateral resistance training No protein, soy protein isolate vs. whey protein isolate	0 g, 20 g, or 40 g of protein	MPS	Relationship between protein intake and MPS are dose and protein source-dependent under rested and post-exercise conditions	[53]
9 months	Men and women Mean age 23 *n* = 147	Resistance training Carbohydrate vs. whey vs. soy protein supplementation	Carbohydrate 1.1 g/kg; Whey 1.4 g/kg; Soy 1.4 g/kg	Body composition; Plasma amino acid	Whey gained more lean mass than soy and carbohydrate	[57]
8 weeks	Men Mean age 21 *n* = 24	Resistance training Rice vs. whey protein isolate	48 g	Body composition, strength and power	No difference observed	[58]
9 weeks	Men Mean age 20 *n* = 18	Resistance training Whey vs. soy protein	33 g protein/day	Lean body mass	Whey gained more lean mass than soy	[59]
6 weeks	Trained men and women 18–35y *n* = 27	Resistance training Whey vs. soy protein vs. maltodextrin	1.2 g/kg body weight	Lean body mass, strength	No difference observed	[60]
12 weeks	Men Mean age 58 *n* = 19	Resistance training	Mixed diet vs. lacto-ovo-vegetarian	Lean body mass; skeletal muscle mass	Mixed diet gained more lean mass than lacto-ovo-vegetarian	[62]
2 weeks	Men Mean age 65 *n* = 21	Resistance training	Lacto-ovo-vegetarian vs. beef containing diet	Muscle size and muscle strength	No difference observed	[63]
5 h	Men Mean age 22 years *n* = 24	EAAs supplementation	6.25 g of protein supplemented with various dose of EAAs	MPS	MPS response of 6.25 g of protein with additional leucine is similar of that 25 g of whey protein	[64]
2 weeks	Men and women Mean age 68 *n* = 8	Leucine supplementation	4 g of leucine/meal; 3 meals/day	MPS	Leucine supplementation increased MPS	[65]
4 h	Men Mean age 66 *n* = 16	Soy protein vs. Soy protein + BCAA	26.5 g of whey and Soy protein meal; equalized BCAA content to casein	Whole body protein synthesis	Spiked soy protein significantly favored whole body protein synthesis	[66]
3 months	Men Mean age 71 *n* = 30	Leucine supplementation	7.5 g/day	Body composition, strength, whole body insulin sensitivity, lipid profile	Supplemental leucine did not improve skeletal muscle mass or strength and does not improve glycemic control or blood lipid profile	[67]
6 months	Diabetic men Mean age 71 *n* = 60	Leucine supplementation	7.5 g/day	Body composition, strength, whole body insulin sensitivity, lipid profile	Supplemental leucine did not improve skeletal muscle mass or strength and does not improve glycemic control or blood lipid profile	[68]
10 days	Men and women Mean age 70 *n* = 25	Bed rest Placebo vs. EAA supplementation	15 g of EAA supplementation	Lean body mass, MPS, muscle function	EAA supplementation improved muscle preservation under bed rest	[69]
3 months	Women Mean age 80 *n* = 75	Exercise vs. exercise + EAA supplementation vs. EAA supplementation vs. health education	3 g of EAA supplement	Body composition, muscle strength and walking ability	Combination of exercise and amino acid supplementation improves muscle strength, mass, and walking ability	[70]
12 weeks	Resistance trained men Mean age 22 *n* = 161	Carbohydrate vs. Pea protein (Nutralys^®^) vs. whey protein concentrate	25 g protein	Body composition; strength	Pea protein and whey protein significantly better than placebo	[71]
8 weeks	High intensity functional trained men Mean age 38 *n* = 8	Whey vs. Pea protein (True Nutrition, Vista, CA, USA)	25 g protein	Body composition, strength	Pea protein and whey protein result in similar body composition and strength	[72]

MPS, Muscle Protein Synthesis; LOV, Lacto-ovo-vegetarian; EAA, essential amino acids.

**Table 2:** Associations of dietary source of protein with measures of muscle in epidemiological studies.

Study Design	Population, *n*	Exposure or Intervention	Outcome Measures	Results	References
Cross-sectional	Men and women; mean age 59; *n* = 2675	Total, animal, plant protein intake; FFQ	Leg lean mass, isometric quadriceps strength	Total and animal protein positively associated with lean mass; Higher quadriceps strength in higher quartiles compared to lowest quartile of plant protein intake	[107]
Longitudinal (6 years)	Men and women; mean age 59; *n* = 5124	Total, animal and plant protein intake; FFQ	Grip strength, arm lean mass	Total and animal protein intake were protective against loss of grip strength	[108]
Longitudinal (3 year)	Men and women; mean age 40; *n* = 2986	Total protein intake, cluster analysis; FFQ	Appendicular lean mass, quadriceps strength	Total protein intake associated with appendicular lean mass and quadriceps strength	[109]
Longitudinal (4 years)	Men and women; mean age 72; *n* = 3122	Net Endogenous Acid Production from Diet; FFQ	Appendicular lean mass	Lower acid load (more plant protein) associated with slower decline in muscle mass	[110]
Longitudinal (4 years)	Men and women; mean age 72; *n* = 2726	Total, animal, plant protein intake; FFQ	Appendicular lean mass	Plant protein intake protective against loss of appendicular lean mass but not total or animal protein	[111]
Longitudinal (2.6 ± 0.4 years)	Female; mean age 62; *n* = 740	Total protein intake; FFQ	Appendicular lean mass, knee extensor strength	Failing to meet the recommended consumption of protein associated with significantly lower ALM, but not muscle strength	[76]
Longitudinal (5 year)	Men and women; mean age 74; *n* = 1561	Total, animal, plant protein intake; FFQ	Total lean mass, appendicular lean mass	Total and animal protein intake is associated with preservation of lean body mass	[77]
Longitudinal (3 year)	Women; mean age 68; *n* = 552	Baseline total protein intake; 3-day food record	Physical performance measures	Higher consumption of dietary protein is associated with better physical function and muscle strength	[75]

FFQ, Food Frequency Questionnaire; ALM, Appendicular Lean Mass.

## Data Availability

This paper do not report any data hence, exlude this statement.
